# Rice Cultivars Under Salt Stress Show Differential Expression of Genes Related to the Regulation of Na^+^/K^+^ Balance

**DOI:** 10.3389/fpls.2021.680131

**Published:** 2021-08-13

**Authors:** Muhammad Farooq, Jae-Ryoung Park, Yoon-Hee Jang, Eun-Gyeong Kim, Kyung-Min Kim

**Affiliations:** Division of Plant Biosciences, School of Applied Biosciences, College of Agriculture and Life Science, Kyungpook National University, Daegu, South Korea

**Keywords:** Na^+^/K^+^homeostasis, cultivar Pokkali, cultivar IR28, high-affinity K^+^ transporter family, sodium/proton exchangers family, salt overly sensitive

## Abstract

Soil salinity is a major problem in agriculture because high accumulation of Na^+^ ions in plants causes toxicity that can result in yield reduction. Na^+^/K^+^ homeostasis is known to be important for salt tolerance in plants. Na^+^/K^+^ homeostasis in rice (*Oryza sativa* L.) involves nine high-affinity K^+^ transporter (HKT) encoding Na^+^-K^+^ symporter, five OsNHX Na^+^/H^+^ antiporters, and OsSOS1 Na^+^/K^+^ antiporter genes. In the present study, we investigated various molecular and physiological processes to evaluate germination rate, growth pattern, ion content, and expression of *OsHKT, OsNHX*, and *OsSOS1*genes related to Na^+^/K^+^ homeostasis in different rice genotypes under salt stress. We found a significant increase in the germination percentage, plant vigor, Na^+^/K^+^ ratio, and gene expression of the OsHKT family in both the roots and shoots of the Nagdong cultivar and salt-tolerant cultivar Pokkali. In the roots of Cheongcheong and IR28 cultivars, Na^+^ ion concentrations were found to be higher than K^+^ ion concentrations. Similarly, high expression levels of *OsHKT1, OsHKT3*, and *OsHKT6* were observed in Cheongcheong, whereas expression levels of *OsHKT9* was high in IR28. The expression patterns of *OsNHX* and *OsSOS1* and regulation of other micronutrients differed in the roots and shoots regions of rice and were generally increased by salt stress. The OsNHX family was also expressed at high levels in the roots of Nagdong and in the roots and shoots of Pokkali; in contrast, comparatively low expression levels were observed in the roots and shoots of Cheongcheong and IR28 (with the exception of high *OsNHX1* expression in the roots of IR28). Furthermore, the *OsSOS1* gene was highly expressed in the roots of Nagdong and shoots of Cheongcheong. We also observed that salt stress decreases chlorophyll content in IR28 and Pokkali but not in Cheongcheong and Nagdong. This study suggests that under salt stress, cultivar Nagdong has more salt-tolerance than cultivar Cheongcheong.

## Introduction

Rice is one of the most important staple foods for a least half the world's population and is considered a salinity-sensitive crop (Munns and Tester, 2008). Rice cultivation systems are threatened by the effects of climate change because many rice-growing areas are located in vulnerable regions (Masutomi et al., 2009). In particular, salinity hazards are major problems in arid and semi-arid regions; irrigation is important for crop production in these regions and the main causes of salinity are salt-rich irrigation water, and improper management of irrigation (Plaut et al., 2013). Salinity stress can result in a 50% yield loss; such a loss was previously estimated at around 6.9 dsm^−1^ for rice (Van Genuchten and Gupta, 1993).

Plants can respond to various environmental stresses on an individual cellular level or synergistically as a whole organism. Salinity stress reduces plants growth rates and can be distinguished by measuring effects immediately upon addition of salt or after several days to weeks (Roy et al., 2014). The cytosol of plant cells normally contains 100–200-mM K^+^ and 1–10-mM Na^+^; this Na^+^/K^+^ quotient is a requisite for many cellular metabolic activities. Cytosolic Na^+^ ion homeostasis may be maintained by transporter genes such as *OsHKT1, OsHKT2*, and *OsVHA* in *Oryza sativa* L. *indica* cvs Pokkali and BRRI Dhan29 (Kader et al., 2006). In *Arabidopsis thaliana*, overexpression of vacuolar Na^+^/H^+^ antiporter can promote plant growth and development in soil when it is watered with up to 200-mM sodium chloride (Apse et al., 1999).

In a previous study, it was proposed that a salt-stress-elicited calcium signal activates a protein kinase complex consisting of the myristoylated calcium-binding protein salt overly sensitive (*SOS)3* and the serine/threonine protein kinase *SOS2*; this protein kinase complex then phosphorylates and activates the plasma membrane Na^+^/H^+^ antiporter *SOS1* (Zhu, 2003). The susceptibility of rice to salinity stress varies with growth stage, but it can cause reductions in final germination percentage, speed of germination, germination energy percentage, and lead to decreased root and shoot lengths and reduced dry matter (Ologundudu et al., 2014).

**Figure 1:**
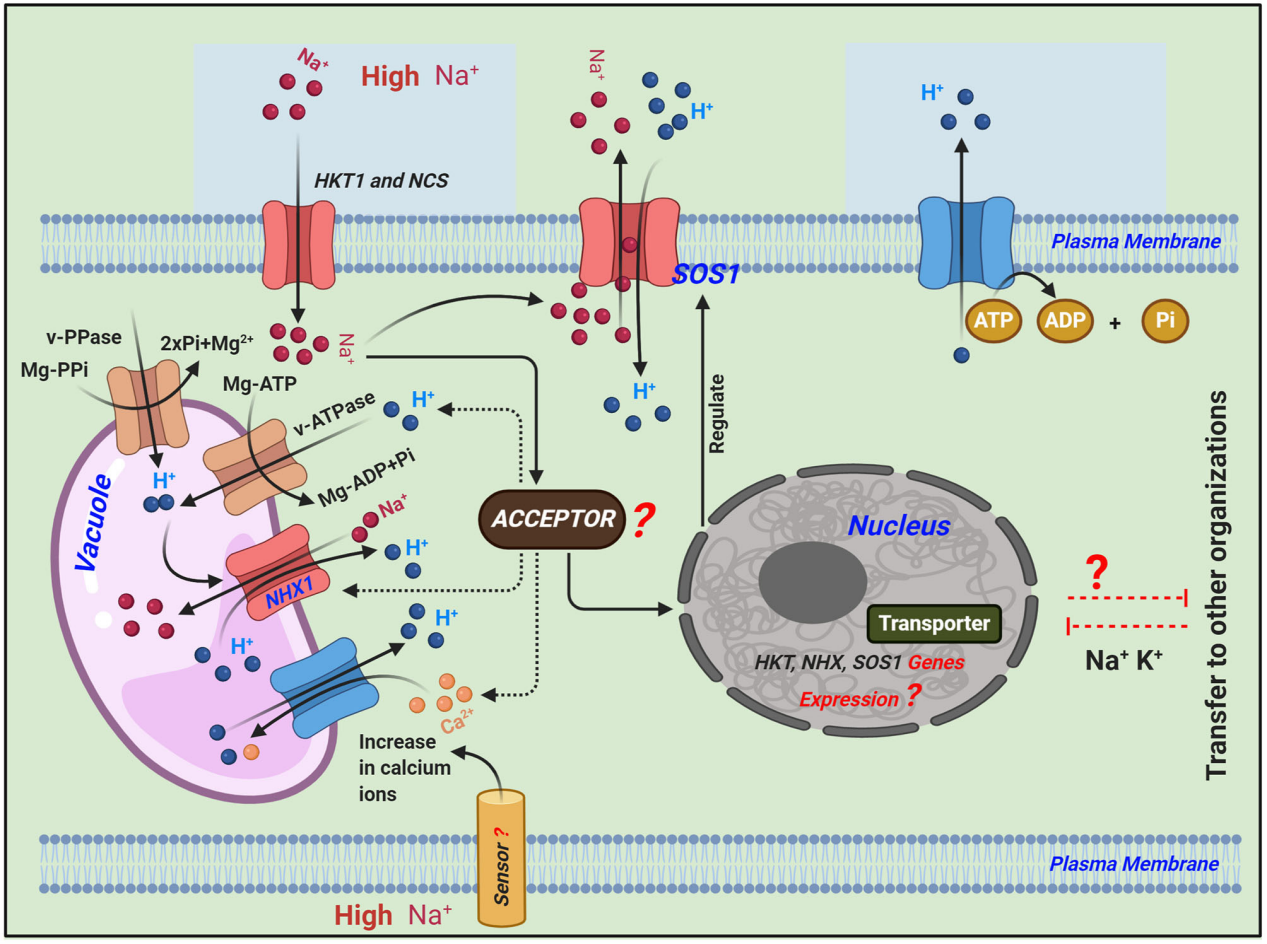
Schematic representation of the transport regulatory mechanisms of Na^+^ in plants under salinity stress adapted from (Roy et al., 2014; Zhang et al., 2018). Different signaling pathways regulate the expression of ion homeostasis-related genes: high-affinity K^+^ transporter (*HKT1*) plays a key role in maintaining Na^+^ homeostasis and mediating Na^+^-specific transport or Na^+^-K^+^ transport. Salt overly sensitive 1 (*SOS1*) plasma membrane Na^+^/H^+^ antiporter exports intracellular Na^+^ to the extracellular space. Sodium/proton exchangers (*NHX1*) in the tonoplast can exchange Na^+^ for protons, resulting in the removal of ions from the cytosol into the vacuole or extracellular space, which thereby minimizes cytotoxicity. While balancing the Na^+^ in the cytoplasm, both of these Na^+^/H^+^ antiporters, i.e., *SOS1* and *NHX1*, convert H^+^ in the extracellular and vacuolar spaces to the cytoplasm, and excess H^+^ in the cytoplasm is transported to the extracellular space via the consumption of energy (ATP is converted to ADP + Pi).

The *O. sativa* cultivar Nipponbare possesses nine *HKT* genes in its genome, with two considered pseudogenes (Garciadeblás et al., 2003). These transporters have also been identified in *Arabidopsis* (Mäser et al., 2002), wheat (Schachtman and Schroeder, 1994; Gassmann et al., 1996), common ice plant (AAK52962) (Su et al., 2003), eucalyptus (Liu et al., 2001), and rice (Horie et al., 2001; Golldack et al., 2002). The salt tolerance of plants may depend on *HKT* transporters, which play critical roles in regulating Na^+^ homeostasis because they mediate Na^+^-specific or Na^+^-K^+^ transport (Garciadeblás et al., 2003). The antiporter genes *OsNHX1* to *OsNHX5* have also been identified in rice (Fukuda et al., 2011). Across the plasma membrane, Na^+^/H^+^ antiporters catalyze the exchange of Na^+^ for H^+^ that regulates internal PH, cell volume, and sodium levels in the cytoplasm. Antiporters are mainly found in yeast, bacteria, animals, and plants with localization typically in the plasma membrane (Orlowski and Grinstein, 1997) and organelles including the perivacuolar compartment (Nass and Rao, 1998) (Figure 1).

Na^+^/K^+^-ATPase or Na^+^-ATPase are absent from plant cells but act to omit Na^+^ from animal and fungal cells (Axelsen and Palmgren, 2001). In plants, H^+^-ATPase and H^+^-inorganic pyrophosphatase are the primary active transporters that induce an H^+^-motive force, whereas the Na^+^/H^+^ antiporter is the main transporter of Na^+^ (Hasegawa et al., 2000). Maintenance of intracellular ion concentrations is essential for ion homeostasis; under salt stress, Na^+^ can enter into plant cells via several pathways and may become harmful to cytosolic enzymes at high concentrations. Therefore, it is essential that plant cells maintain high and low K^+^ and Na^+^ concentrations in the cytosol, respectively, and that they release excessive Na^+^ or collect it in vacuoles (Taiz and Zeiger, 2002). In *A. thaliana*, Na^+^/H^+^ antiporters, encoded by the *AtSOS1* gene, help to catalyze Na^+^ efflux in the plasma membrane (Shi et al., 2000, 2002).

The solubility of micronutrients such as (Cu, Mn, Zn, Fe, and Mo) is generally low, in both saline and sodic soil, and plants growing on such soils often experience deficiency of these elements but not in all cases (Page, 1996). Under salt stress, maize is grown both in solution culture, and soil (Izzo et al., 1991; Rahman et al., 1993), show a decrease in the concentration of Cu ion in the leaf and stem parts, but on the other hand salinity stress, substantially increased leaf Cu in hydroponically-grown tomatoes (Izzo et al., 1991). Most of the studies suggested that salt stress decreases Mn level in corn shoot tissue (Izzo et al., 1991; Rahman et al., 1993). However, some studies, show that salinity had no effect (Al-Harbi, 1995) or increase the Mn level in leaf or shoot tissue of tomato (Niazi and Ahmed, 1984). Most of the studies demonstrate that salinity stress, increase Zn concentration in shoot tissue such as in maize (Rahman et al., 1993), tomato (Knight et al., 1992), and citrus (Ruiz et al., 1997). But in some studies, it was not affected (Izzo et al., 1991), or on the other hand, Zn concentration was found to decrease in the case of cucumber leaves (Al-Harbi, 1995). Previous study reported that under salt stress, 12 soybean cultivars show a higher level of Fe, Mn, Cu, and Zn in the region of the roots compared with those in leaves and stem (Tunçturk et al., 2008). In a previous study, it was reported that salinity stress, did not affect on grapevine Shoot Ca and Mg, trunk P, and Mg, and root P, Ca, and Mg concentration. Some studies suggested that salt stress has an antagonistic effect on Ca^+2^, K^+^, Fe, Mn, P, and Zn but has a synergistic effect on Nitrogen (N) and Mg in rice crops (Jung et al., 2009; García et al., 2010).

In the present study, we compared two famous cultivars of the plant molecular breeding lab at Kyungpook National University, South Korea, namely Nagdong and Cheongcheong, with the salt-tolerant variety Pokkali and salt-sensitive variety IR28. We conducted molecular and physiological analyses of these four rice genotypes to identify the relative salt tolerance and sensitivity of the cultivars from the plant molecular breeding lab. The findings of this study could help develop new salt-tolerant or -sensitive cultivars via CRISPR/Cas9 knockout or overexpression methods in future studies.

## Materials and Methods

Four different rice (*O. sativa*) genotypes Pokkali (Gyehwa-20), IR28, Cheongcheong, and Nagdong were provided by the plant molecular breeding lab of Kyungpook National University. Pokkali (Gyehwa-20) is a unique salt-resistant cultivar that is cultivated in water-logged coastal regions, whereas IR28 is salt-sensitive. The cultivars Cheongcheong and Nagdong were used for comparison; “Cheongcheong (IT228761, IT number is a resource number managed by the National Academy of Agricultural Sciences of Rural Development Administration, Korea)” was established in 1974, YR675153-2-2, a sister line of “Miryang 29,” which is a high-quality and -yield, to cultivate a new variety of disease resistance, high quality, safety and high yield. “Nagdong (IT006182)” was an artificial crossing in 1968 with a copy of Mineyudaka, a Japanese-type virus-resistant variety, as a model of “Nonglim 6” at the Yeungnam Agricultural Research Institute (Milyang, Gyungsnagnam-do, Korea) in order to cultivate a variety of safe virus-resistant varieties. These are two famous cultivars of the plant molecular lab from which the double haploid population consists of 133 lines that were derived from 2010 to 2012 (Yun et al., 2014) at Gunwi-gun near Kyungpook National University.

### Seed Germination Test

Rice seeds of the four different genotypes were surface sterilized with 70% ethanol and 1% (v/v) sodium hypochlorite solution for 30 min and afterward rinsed three times with deionized water. Small Petri dishes (~9 cm in diameter) containing autoclaved filter paper were used for germination tests; 15 seeds were placed in each Petri dish to give three replicates for each cultivar. Salt solutions of 0, 100, 150, 200, 300, and 400 mM were used in the germination tests. Each Petri dish was fixed with plastic paraffin film and placed inside a growth chamber with a 12 h light/12 h dark photoperiod at 30°C in the light and 25°C in the dark. Relative humidity was maintained at 60% inside the growth chamber. The salt stress treatment lasted for two weeks. Seeds were considered to have germinated when the radical was protruding from the seed coat. Germinated seeds were counted after each week.

### Growth Conditions and Salt Treatment

Clean and strong seeds of the four different rice cultivars were selected for germination and incubated for 4 days at 33°C using small plastic bags previously punched with a screw to allow entry and exist of water inside the incubator. Uniformly germinated seeds were selected and cultivated for 5 weeks in pots containing soil in a greenhouse at 20–25°C with a 10 h light/14 h of dark photoperiod to give three replicates for each cultivar. Before salinity stress, soil clay was kept overnight, and afterward filter the clay soil using filter paper and measure pH 6.5 using a pH meter for 1 kg of soil. After 5 weeks, we treated the rice cultivars with 150 mM NaCl for 7 days. The rice plants were watered daily with 150 mL of brine solution. After salinity stress, the rice plants were washed five times with tap water and three-time with distilled water afterward kept for 30 min in 150 mM NaCl solution. The samples were dried at 65°C for 2 days and determined the ion contents in both roots and shoot regions, and phenotypic data were also collected before and after the 7 days of salinity stress.

### Measurement of Chlorophyll Content

Chlorophyll content was measured after salinity stress in control and treated cultivars using a SPAD device (Konica Minolta Sensing, Inc. Japan). From each replicate, five leaves were randomly selected and the lower apex, middle, and lower portions of the leaves were used to determine chlorophyll content.

### Quantitative Real-Time PCR Analysis

Twenty germinating seeds of each rice genotype were grown for 14 days in pots containing soil up to the three-to-four-leaf stage. These plants were treated with 150-mM NaCl and then RNA was extracted from their roots and shoots regions after 0, 8, 24, and 48 h using an RNeasy Plant Mini Kit (Qiagen, Germany) according to the manufacturer's instructions. A NanoDrop 2000 spectrophotometer (Thermo Scientific, Wilmington, DE, USA) was used to measure RNA concentrations. For first-strand cDNA synthesis, the qPCRBIO cDNA Synthesis Kit and 400 ng of total RNA were used. For quantitative RT-PCR, we used the Eco Real-Time PCR system (Illumina, Inc., San Diego, CA, USA), 2X qPCRBIO SyGreen (www.pcrbio.com; London, UK), and primers specific to the selected genes (Table 1). *OsActin1* (accession no. AB047313) was used as an internal reference gene for normalization.

**Table 1:** List of primers used for qRT-PCR analysis of different rice genotypes.

**Accession**	**Gene**	**Forward primer (5^′^-3^′^)**	**Reverse primer (5^′^-3^′^)**	**Fragment size (bp)**
AB061311	OsHKT1	TCGGCAAGCACTGTGATAAG	CGCTTGCTCCTCTTCAAATC	98
AB061313	OsHKT2	GGGAAAGGTGACCAAGTTGA	AGTCGGCAACTTAGGAAGCA	91
AJ491820	OsHKT3	AACAGCAGCGCAGTAGGTTT	CAACCTCCACAACTGCAAGA	73
AJ491816	OsHKT4	AACTGGGTCCTTTTGCTCCT	ACCTTCCCCAAAACCCATAC	98
AJ491818	OsHKT6	GGAAGCACCCAATACATGCT	AATGTGCGGAAAGTTTGGAG	100
AK109852	OsHKT7	CATCTGCATCACCGAGAGAA	CTTGCCTGACAACTTCGACA	86
AK108663	OsHKT8	CTCAGGGAAGTGGAGCAAAC	AACTTCTTGAGCCTGCCGTA	72
AJ491855	HKT9	ATTCCATCTTAGCCCGCTTT	CCAAGGCAACAAAACCAAGT	74
AB021878	OsNHX1	ATTGGGGAATCTGTTTGCTG	ACAGACAGCTAGGCCCAGAA	84
AB531435	OsNHX2	GCTATTCAACGCAATGCAGA	GTGCTCGTGGCAAACAGATA	99
AB531433	OsNHX3	ATGGATGCACTGGACATTGA	TGAATTGGTCGTGGACAAAA	70
AB531434	OsNHX5	GATGGACCTGGGCTACAGAA	AGATGGGCAATGGAAACAAG	98
AY785147	OsSOS1	GGCAGGATAATGTGGTGCTT	TGAGCAGCAGGCAATATCAC	70
AB047313	OsActin1	CGTCCTCCTGCTTGTTTCTC	TAGGCCGGTTGAAAACTTTG	72

### Analysis of Ion Content

Roots and shoots of different rice genotypes from the greenhouse were used for ion determination as described by Rus et al. (2001). Briefly, samples were dried at 65°C for 2 days, and then ground in liquid nitrogen. Subsequently, 100 mg of tissue powder was extracted with 10 mL of 0.1 N HNO3 for 30 min. Samples were filtered and Na^+^/K^+^ ions and other micronutrients were analyzed using an ICP Spectrometer (I) (Optima 7300DV & Avio500; PerkinElmer).

### Statistical Analyses

Statistical analysis was performed for three replicates, where each replication was considered as a block and arranged in different Petri dishes or pots in the control conditions. The experiment was repeated twice. Differences among treatment means were evaluated using Duncan's multiple range test with significance set at *P* < 0.05. Data analysis was conducted in SPSS (IMMSPSS Statistics, version 22, NC). Figures were produced using Graph Pad Prism version 5.0 (Graph Pad Software Inc., San Diego, USA).

## Results

### Salinity Effect on Seed Germination

**Figure 2:**
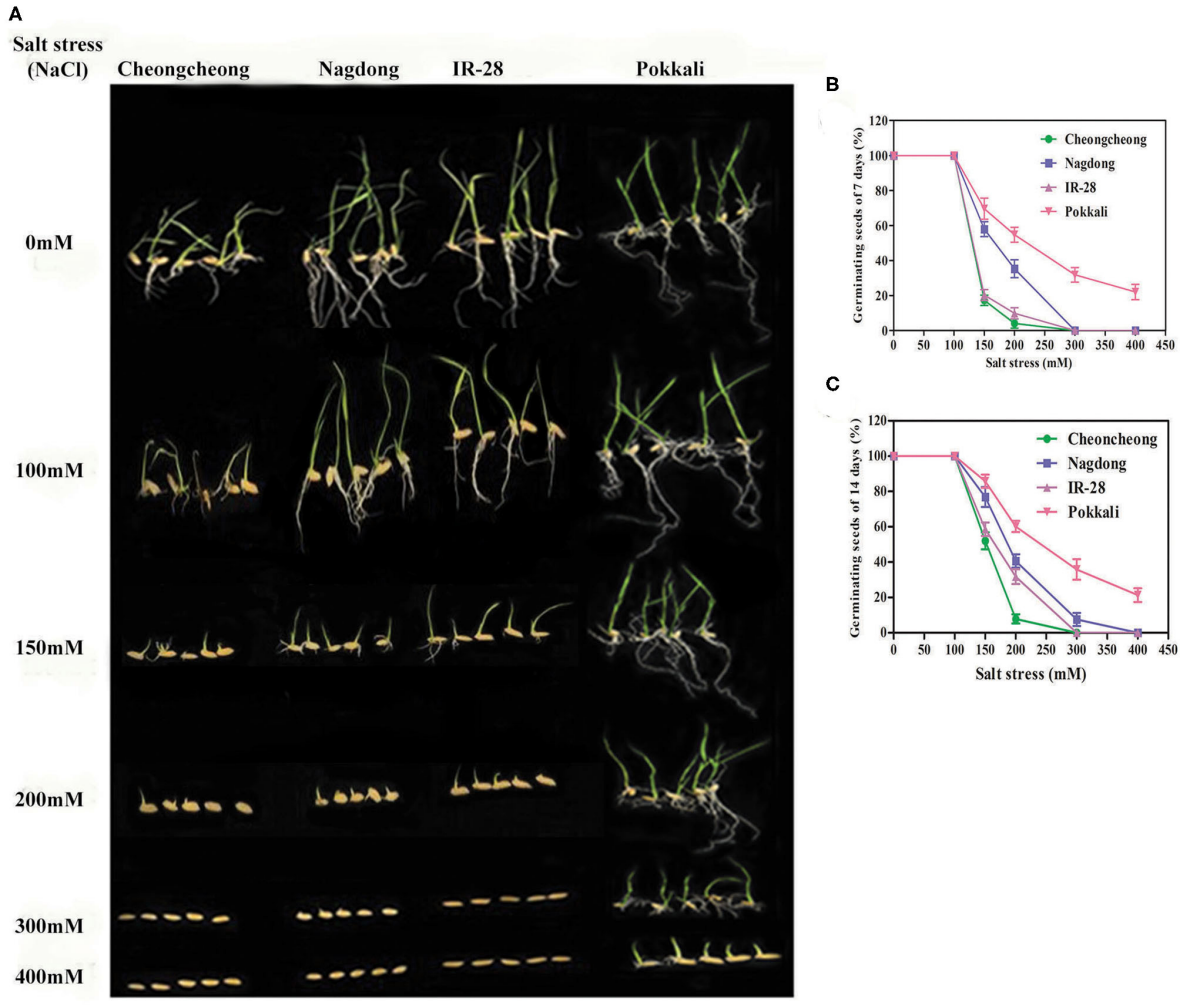
Seed germination rate and seedling growth of different rice genotypes under salt stress. Various concentrations of salt stress were used to determine the seed germination and seedling growth represented in **(A)**, **(B)**, and **(C)**.

Among the four different rice genotypes, germination percentage and growth of rice seedlings gradually decreased as salt concentration increased (Figures 2A–C). After 7 days of salt stress, there was a significant increase in the percentage of seeds germinating in both Pokkali and Nagdong up to 200-mM NaCl. A few seeds germinated under 300-mM NaCl in Nagdong, but at 400-mM NaCl Nagdong seeds failed to germinate. Seed germination was significantly higher in Pokkali than in the other three rice genotypes. Seed germination percentage was significantly higher in the salt-sensitive cultivar IR-28 at 200-mM NaCl than that in Cheongcheong, but IR-28 seed germination was lower than Nagdong seed germination (Figure 2B). In both Cheongcheong and IR-28, seed germination was completely inhibited in the presence of 300 and 400 mM of NaCl with 7–14 days of treatment. However, more than 20% of seeds germinated in Pokkali with salt stress of 400 mM NaCl (Figures 2B,C).

### Growth and Chlorophyll Reduction Under Salinity Stress

**Figure 3:**
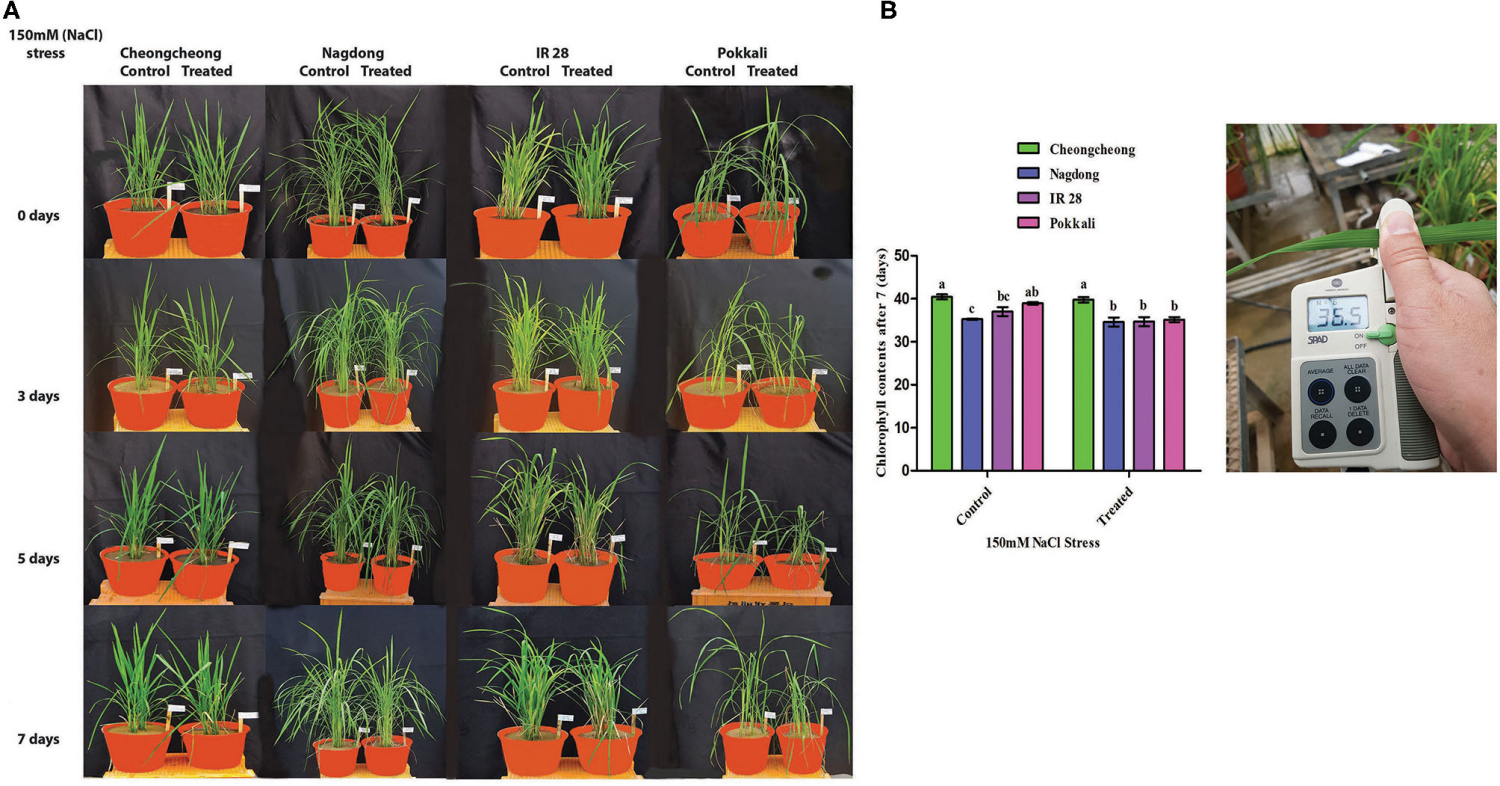
Phenotypic representation of different rice genotypes under 150-mM salinity stress in a greenhouse. **(A)** Effects of salt stress after 3, 5, and 7 days; **(B)** Chlorophyll measurements before and after salinity stress.

Growth was affected in all rice genotypes after 3, 5, and 7 days of salinity stress. The growth rate of all rice cultivars declined under 150-mM NaCl stress relative to the growth in the control (Figure 3). The growth rates of Cheongcheong, IR28, and Pokkali dramatically alter compared with growth in the control. However, Nagdong suffered a slight in growth under salinity stress when compared with salt-treated Cheongcheong, IR28, and Pokkali cultivars (Figure 3). Similarly, under salinity stress, the chlorophyll content of IR28 and Pokkali were significantly reduced compared with that in the control group. The highest chlorophyll content was recorded in Cheongcheong before and after salinity stress; similarly, Nagdong maintained its chlorophyll content under salt treatment. The chlorophyll contents of Cheongcheong, IR28, and Pokkali were significantly higher than that of Nagdong in the control groups; however, after salinity stress, the chlorophyll contents of all cultivars were statistically similar, except for those of Cheongcheong (Figure 3B).

### Comparison of Ion Transport-Related Gene Expression Under Salt Stress Among Rice Genotypes

**Figure 4:**
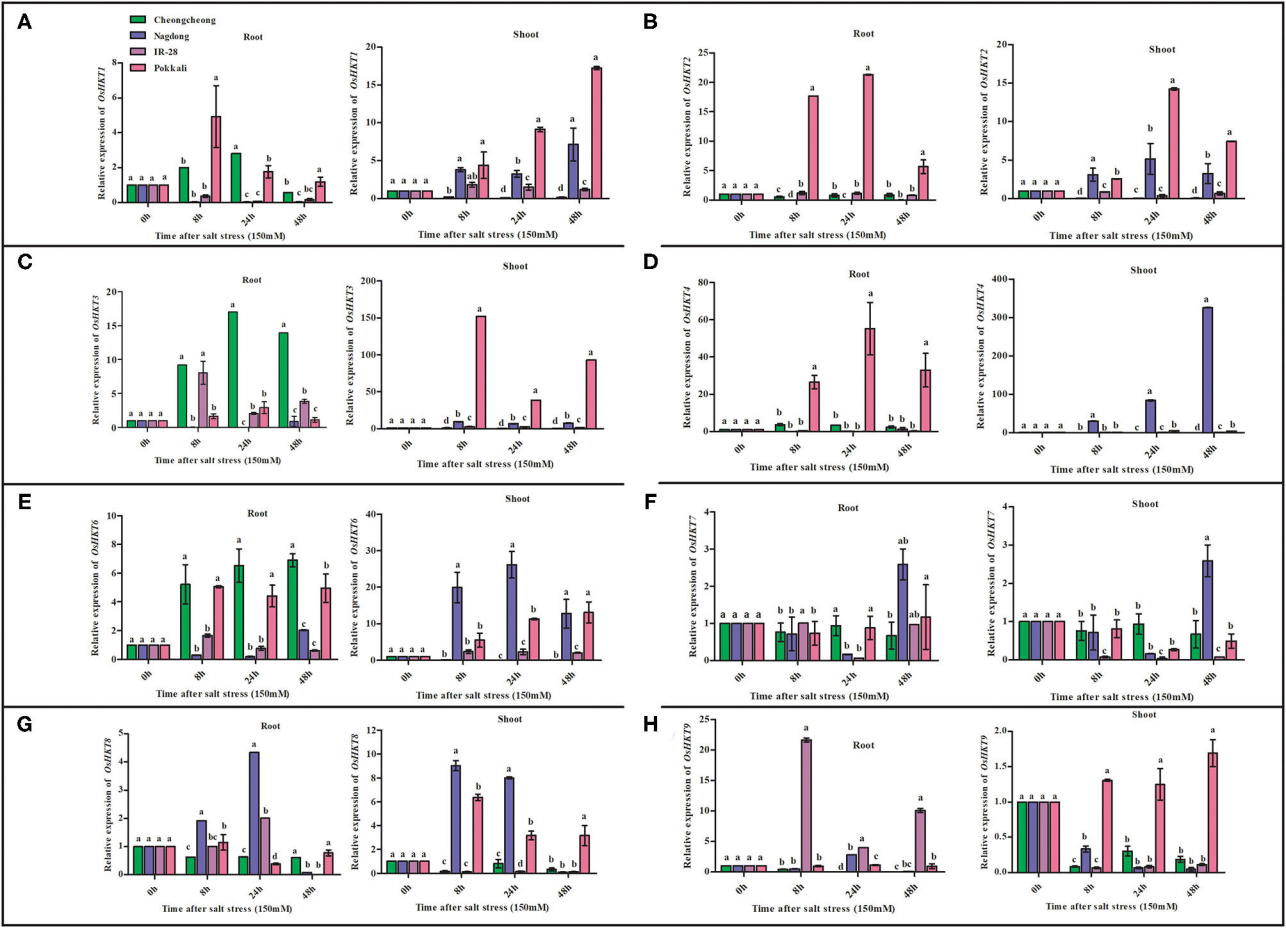
Quantitative real-time PCR analyses of *HKT* family genes from root and shoot tissues of salt-tolerant and salt-sensitive rice varieties (Pokkali and IR28) and plant molecular breeding lab varieties (Cheongcheong and Nagdong) under salt stress. The expression pattern of *OsHKT* genes under 150-mM NaCl stress is shown in **(A–H)**. Different letters in the graphs indicate statistical differences among the treatments when compared with the control (*P* < 0.05 by Duncan's multiple range test).

The expression levels of eight ion transport regulation genes differed under salt stress according to real-time PCR (Figure 4). Among eight *HKT* genes, *OsHKT1, OsHKT2, OsHKT3, OsHKT4, OsHKT6*, and *OsHKT9* were highly expressed in the roots and shoots regions of salt-tolerant cv. Pokkali (Figures 4A–E,H). *OsHKT8* was highly expressed only in the shoots regions (Figure 4G). After salt stress treatment, *OsHKT* genes were differentially regulated in Pokkali rice; however, high expression of these genes was mainly found at 8 and 24 h. Similarly, *OsHKT1, OsHKT3*, and *OsHKT6* were upregulated in the root region of Cheongcheong at 8, 24, and 48 h (Figures 4A,C,E). However, all *OsHKT* family genes were downregulated in the Cheongcheong shoot regions at 8, 24, and 48 h (Figures 4A–H). The Nagdong cultivar showed the most significant upregulation of *OsHKT1*, OsHKT3, *OsHKT4*, and *OsHKT7* at 48 h in the shoot regions (Figures 4A,C,D,E). In contrast, *OsHKT6, OsHKT7*, and *OsHKT8* genes were upregulated at 8, 24, and 48 h in both the roots and shoots regions of Nagdong (Figures 4E–G). All *OsHKT* family genes were significantly downregulated in both the roots and shoots regions of salt-sensitive IR-28 except for *OsHKT3* and *OsHKT9*, which were significantly upregulated in the roots region at 8, 24, and 48 h (Figures 4C,H).

**Figure 5:**
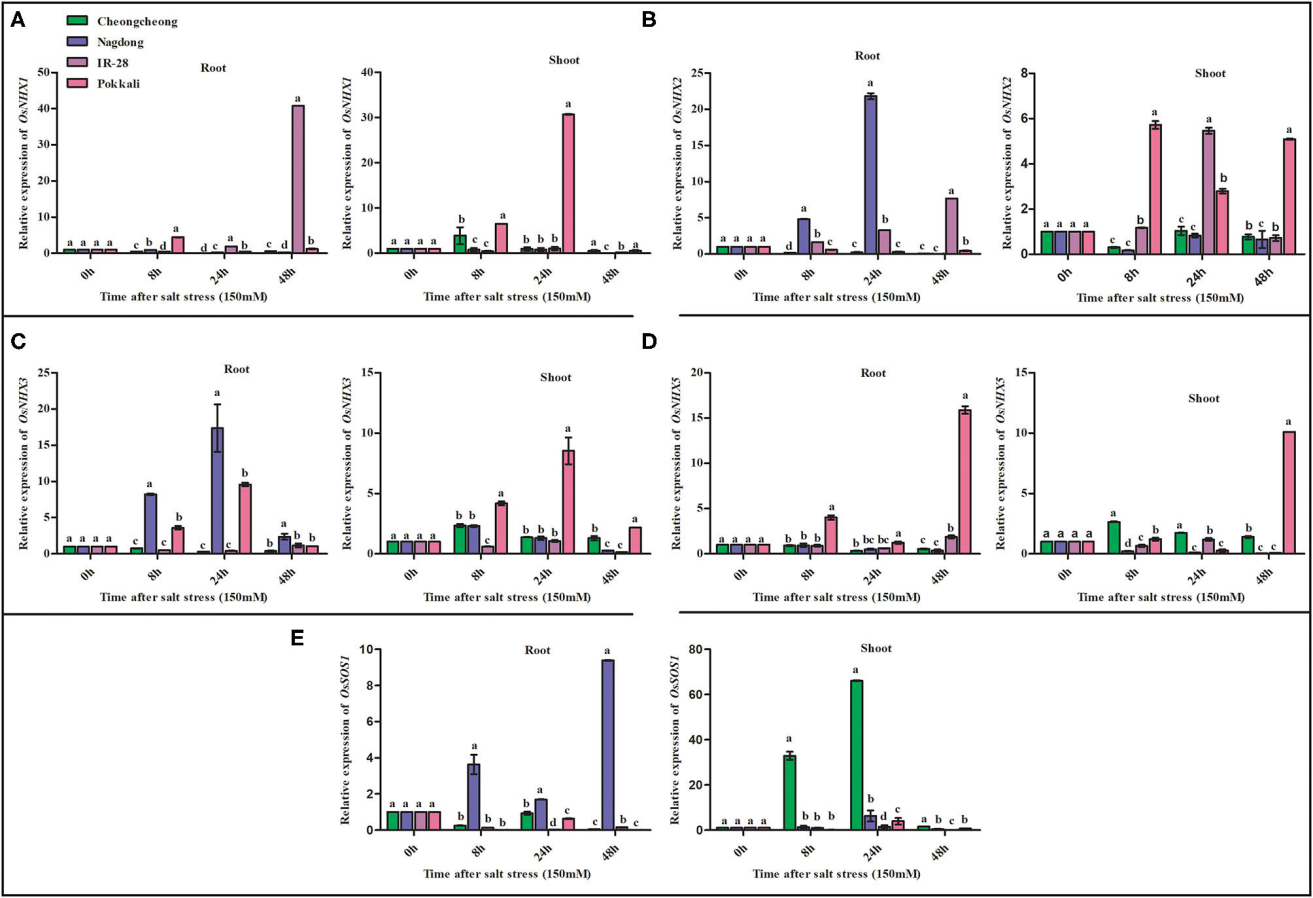
Quantitative real-time PCR expression analysis of *NHX* family and *OsSOS1* genes from the root and shoot tissues of different rice genotypes. The expression patterns of *OsNHX* and *OsSOS1* genes under 150-mM NaCl stress are shown in **(A–E)**. Different letters in the graphs indicate statistical differences among the treatments when compared with the control group (*P* < 0.05 by Duncan's multiple range test).

Under salt stress treatment, OsNHX family genes were differentially expressed at different time points. *OsNHX1* was significantly upregulated in both IR-28 and Pokkali, with maximum levels of expression observed at 48 h in the roots of IR-28 and at 24 h in the shoots of Pokkali (Figure 5A). Similarly, *OsNHX2* was upregulated in both IR-28 and Pokkali, but high expression levels were observed at 8, 24, and 48 h in the shoots of Pokkali and at 24 h in IR-28 (Figure 5B). High expression of *OsNHX2* was observed in the roots region of Nagdong at 8 and 24 h and IR-28 at 8, 24, and 48 h (Figure 5B). The expression of *OsNHX3* was upregulated in the roots of Nagdong and Pokkali at 8 and 24 h; in the shoots region, it was only upregulated in the Pokkali cultivar (Figure 5C). High expression of *OsNHX5* was observed in both the roots and shoots regions of Pokkali at 48 h of stress (Figure 5D). On the other hand, *OsNHX5* was highly expressed in the shoots of Cheongcheong at 8, 24, and 48 h of salt stress (Figure 5D). In contrast, expression of *OsSOS1* was significantly downregulated in the roots and shoots of both IR-28 and Pokkali; however, *OsSOS1* was significantly upregulated in the roots and shoots of both Nagdong and Cheongcheong at 8, 24, and 48 h (Figure 5E).

### Comparative Analysis of Ion Content Under Salt Stress

Under salt stress, we examined nine elements, namely Na^+^, K^+^, Ca^2+^, Mg^2+^, Cu^2+^, Zn^2+^, Fe^2+^, Mn^2+^, and P, in the roots and shoots of both control and treated plants. These elements were differentially regulated in the rice genotypes under salt stress. After 7 days of 150-mM salt stress, Na^+^, and K^+^ ions were inversely regulated in the roots and shoots of control and treated plants: as Na^+^ levels increased K^+^ ions decreased and vice versa. Under salt stress, Na^+^ content significantly increased in the roots of all four rice genotypes relative to levels in the control, whereas K^+^ content decreased in the roots of Cheongcheong and Pokkali relative to content in the control (Table 2). On the other hand, in Nagdong and IR28, K^+^ content was significantly higher than that in the control. In the respective roots of Cheongcheong and IR28, content of Na^+^ increased by ~748.35- and 2140.06-fold, whereas K^+^ ion content inversely decreased by 698.45- and 1443.72-fold. In Nagdong and Pokkali roots, K^+^ content increased by ~1069.88- and 3786.88-fold, whereas Na^+^ content decreased by 935.50- and 3556.27-fold, respectively. Both Na^+^ and K^+^ contents were significantly higher in salt-treated plant shoots than in control plant shoots. In treated plant shoots, Na^+^ and K^+^ ions were present inversely, with K^+^ ions found at significantly higher levels than those of Na^+^ ions in all four rice genotypes. The content of K^+^ ions was approximately 406.82-, 2684.84-, 6365.42-, and 7562.18-fold higher than that of Na^+^ ion in the treated cultivars Cheongcheong, IR28, Nagdong, and Pokkali, respectively (Table 2.). In addition to Na^+^ and K^+^ ions, the uptake and transportation of other ions also differed in the roots and shoots of all four rice genotypes relative to that in the control. For example, compared with control plants, the content of Ca^2+^ decreased after salt stress in the roots of Nagdong and shoots of Pokkali, whereas Mg^2+^ content decreased in the roots of Cheongcheong. Both Ca^2+^ and Mg^2+^ content were increased in the roots and shoots of IR28, as well as in the shoots of Cheongcheong and Nagdong, and in the roots of Pokkali; in the shoots of Pokkali, only Mg^2+^ was increased. In addition, Cu^2+^ content increased in the roots and shoots of all rice genotypes after salt stress except in IR28 shoots. On the other hand, after salt stress and compared with the control, Zn^2+^ content increased in the roots and shoots of Nagdong and IR28, but was significantly decreased in the roots and shoots of Cheongcheong and Pokkali. Furthermore, the contents of Fe^2+^, Mn^2+^, and P were significantly increased in Cheongcheong and Nagdong shoots. In the roots of Cheongcheong, Fe^2+^ and P decreased, and in the roots of Nagdong Mn^2+^ decreased, all relative to levels in control plants. In the roots of IR28, the content of Fe^2+^, Mn^2+^, and P were increased; in the roots of Pokkali, these three elements were significantly decreased. However, in the shoots of IR28, Mn^2+^ was increased; in the shoots of Pokkali, Fe^2+^ and P were increased.

**Table 2:** Inductively coupled plasma spectroscopy analysis [in ppm (mg/kg)] conducted under 150-mM NaCl stress.

	**Cheongcheong**				**Nagdong**			
**Ions**	**Control**	**Treated root**	**Control**	**Treated shoot**	**Control**	**Treated root**	**Control**	**Treated shoot**
	**root**	**150 mM (NaCl)**	**shoot**	**150 mM (NaCl)**	**root**	**150 mM (NaCl)**	**shoot**	**150 mM (NaCl)**
Na^+^	7162.76 ± 167.75^a^	13566.1 ± 748.35^bc^	886.88 ± 6.43^d^	9438.99 ± 104.51^cd^	2331.19 ± 30.89^b^	16045.18 ± 935.5^b^	817.76 ± 56.51^d^	11543.04 ± 2034.33^bcd^
K^+^	47322.37 ± 1492.07^a^	13311.39 ± 698.45^bc^	15703.47 ± 240.86^d^	32020.11 ± 406.82^a^	7205.56 ± 34.78^e^	17962.58 ± 1069.88^b^	5852.8 ± 339.86^e^	35696.97 ± 6365.42^a^
Ca^2+^	1216.82 ± 47.38^f^	2128.53 ± 64.67^f^	1905.42 ± 12.12^d^	3398.85 ± 57.98^b^	4282.03 ± 25.9^a^	3784.76 ± 58.75^a^	1035.65 ± 55.42^g^	2629.04 ± 38.74^e^
Mg^2+^	3718.77 ± 137.94^b^	3129.29 ± 40.79^c^	1010.24 ± 9.95^f^	2611.5 ± 90.12^d^	2795.77 ± 10.81^c^	3542.99 ± 31.88^b^	773.18 ± 46.97^g^	2200.65 ± 44.84^e^
Cu^2+^	53.69 ± 3.01^a^	54.58 ± 1.75^a^	12.9 ± 0.08^d^	27.66 ± 0.14^d^	19.21 ± 0.18^c^	23.55 ± 0.11^f^	6.46 ± 0.45^e^	25.71 ± 0.13^e^
Zn^2+^	109.02 ± 1.8^a^	108.57 ± 3.25^a^	30.43 ± 1.16^e^	63.16 ± 0.28^cd^	66.99 ± 8.6^c^	84.25 ± 1.36^b^	16.26 ± 3.8^f^	58.76 ± 3.05^d^
Fe^2+^	478.08 ± 20.84^d^	466.9 ± 13.22^g^	710.89 ± 0.63^b^	1226.89 ± 14.53^c^	471.12 ± 3.12^d^	800.45 ± 1.4^e^	627.24 ± 35.79^c^	1464.06 ± 6.94^b^
Mn^2+^	2524.12 ± 131.26^c^	7949.05 ± 106.31^a^	269.05 ± 7.81^de^	4223.22 ± 174.5^d^	9425.34 ± 164.28^a^	7646.2 ± 83.68^b^	229.48 ± 48.21^e^	864.62 ± 23.16^f^
P	4115.76 ± 115.19^a^	1596.51 ± 31.11^g^	1816.64 ± 7.19^d^	3616.16 ± 25.89^a^	1616.29 ± 8.25^e^	2931.81 ± 16.46^d^	742.49 ± 57.3^f^	3427.35 ± 4.53^b^
**IR28**					**Pokkali**			
Na^+^	1863.74 ± 117.75^bc^	30116.15 ± 2140.06^a^	1158.76 ± 48.31^cd^	7430.18 ± 952.49^d^	7837.89 ± 1059.42^a^	34243.13 ± 3556.27^a^	1830.72 ± 38.83^bc^	10427.33 ± 2230.61^cd^
K^+^	5271.43 ± 206.6^e^	20496.77 ± 1443.72^b^	17127.61 ± 421.77^d^	19987.48 ± 2684.11^b^	42294 ± 5328.69^b^	37697.25 ± 3786.88^a^	28045.71 ± 11.87^c^	35329.41 ± 7562.18^a^
Ca^2+^	1053.14 ± 46.44^g^	2010.48 ± 25.65^g^	1504.71 ± 16.81^e^	2049.78 ± 14.73^fg^	2586.02 ± 69.3^c^	3017.59 ± 43.75^d^	3256.09 ± 2.43^b^	3153.01 ± 28.96^c^
Mg^2+^	1144.26 ± 54.67^ef^	3513.83 ± 2.51^b^	1242.62 ± 48.19^e^	1538.29 ± 45.18^f^	3907.19 ± 62.24^a^	4482.88 ± 60.05^a^	1833.98 ± 16.14^d^	2099.7 ± 43.35^e^
Cu^2+^	19.02 ± 0.53^c^	31.02 ± 0.04^c^	14.79 ± 0.06^d^	13.34 ± 0.03^h^	26.75 ± 0.39^b^	35.56 ± 0.05^b^	13.9 ± 0.23^d^	16.16 ± 0.18^g^
Zn^2+^	27.92 ± 0.59^e^	64.35 ± 1.51^c^	24.35 ± 0.65^e^	32.94 ± 1.36^e^	87.07 ± 0.51^b^	59.46 ± 2.22^cd^	42.86 ± 1.2^d^	30.74 ± 2.83^e^
Fe^2+^	181.97 ± 0.05^e^	274.86 ± 5.4^h^	708.76 ± 12.18^b^	697.62 ± 1.51^f^	1986.29 ± 45.56^a^	1051.35 ± 5.99^d^	1981.8 ± 15.78^a^	3340.17 ± 64.3^a^
Mn^2+^	2345.94 ± 113.99^c^	3138.09 ± 49.03^e^	220.81 ± 1.05^e^	547.4 ± 205.78^g^	8454.1 ± 268.94^b^	6772.82 ± 76.93^c^	545.7 ± 6.98^d^	218.18 ± 15.52^h^
P	461.38 ± 16.68^g^	1704.81 ± 30.75^f^	1841.56 ± 19.5^d^	1752.12 ± 19.72^f^	2643.12 ± 64.05^b^	2321.95 ± 7.53^e^	2364.91 ± 8.34^c^	2990.55 ± 32.89^c^

*After 7 days of salt stress, Na^+^, K^+^ and other micronutrients, such as Ca^2+^, Mg^2+^, Cu^2+^, Zn^2+^, Fe^2+^, Mn^2+^, and P, were measured in the roots and shoots of both control and treated plants. Data ± standard error are shown. Different letters after data (a–h) indicate significant differences between salt treatments (P <0.05 by Duncan's multiple range test)*.

## Discussion

### Salinity Inhibits Seed Germination and Reduces Rice Seedling Growth

A previous study suggested that salinity stress up to 20 dS m^−1^ strongly inhibits rice seed germination, yield reduction, growth reduction in shoot and root length, and dry matter (Hakim et al., 2010). However, another study reported that rice is salt tolerant to some extent at germination and, in some cases, not significantly affected by salt up to 16.3 dS m^−1^ (Khan et al., 1997). In contrast, high concentrations of salinity affect the seedling stage of rice (Lutts et al., 1996). Our results demonstrate that NaCl, treatment inhibits rice seed germination as well as seedling growth as salt concentrations increase. Furthermore, the high levels of germination were recorded in the salt-resistant Pokkali and plant molecular breeding lab Nagdong cultivars. Salt stress inhibits seed germination along with seedling growth, reduces photosynthesis levels, and promotes senescence (Tuteja et al., 2013). However, up to 100-mM NaCl, the germination percentage was similar among the four tested rice genotypes.

### Salt Accumulation Leads to Reduced Growth Patterns and Chlorophyll Content

Through several different processes, salinity reduces plant growth, which is related to the accumulation of salt in the shoots and/or roots. These effects can be experimentally distinguished within a minute to several days or weeks (Sirault et al., 2009). For example, salt stress reduces the growth of sugar beet (Ghoulam et al., 2002), cotton (Meloni et al., 2001), and tomato (Romero-Aranda et al., 2001). To explore that salinity stress decreases the growth rate and chlorophyll content. In our study, we observed growth reduction in all genotypes under salt stress. High salt uptake mostly causes necrosis and leaf tip burn in plants (Wahome et al., 2001). Previous studies suggest that both growth and photosynthesis are interdependent; therefore, environmental stress affecting growth also affects photosynthesis (Dubey, 1996; Taiz and Zeiger, 1998). Another report has indicated that salt stress reduces photosynthesis rates mainly via water potential (Cushman et al., 1989). Our results demonstrate that, under salinity stress, chlorophyll content was decreased in IR28 and Pokkali compared with the content in Cheongcheong and Nagdong, all relative to control plant levels.

### Differential Expression of Ion Transport-Related Genes in Rice Genotypes Under Salt Stress

We detailed the differential pattern of gene expression of *HKT, NHX*, and *SOS1* in the roots and shoots regions of rice genotypes under salt stress. In the current study, we found high induction of *OsHKT1* in the roots of Cheongcheong and Pokkali following 8 and 24 h of salt stress, although the degree of induction in the shoots of Nagdong and Pokkali was varied with time. However, in salinity stress, Na^+^ ion competition at K^+^ binding sites may contribute to K^+^ deficiency (Maathuis and Amtmann, 1999) and thus might cause the high induction of *OsHKT1* in rice genotypes observed here. Under a high level of NaCl, there is another possibility that excess Na^+^ entering the cytosol increases the ideal cytosolic Na^+^/K^+^ ratio in cells which might recognize as a K^+^ deficiency, thus inducing *OsHKT1* as suggested by Horie et al. (2001) in case of the K^+^ deficiency. A previous study reported that 150-mM NaCl stress induced high levels of *OsHKT1* in both the root and shoot tissues of salt-sensitive BRRI Dhan29, whereas salt-tolerant Pokkali showed high expression of *OsHKT2* in shoots and lower expression in root tissue (Kader et al., 2006). Our results also suggest the involvement of *OsHKT2* in the salt-stress response, especially in salt-tolerant Pokkali. High induction of *OsHKT2* was observed in Pokkali, which was 20-fold higher in the roots, 15-fold higher in the shoots, and 5-fold higher in the shoot of Nagdong.

Previous studies have reported tissue-specific localization of three members of each HKT subfamily: from subfamily 1: AtHKT1;1 from *Arabidopsis* (Berthomieu et al., 2003; Horie et al., 2005), OsHKT1;5 from rice (Ren et al., 2005), and McHKT1 from ice plant (Su et al., 2003); for sub family 2: TaHKTT2;1 from wheat (Schachtman and Schroeder, 1994), OsHKTT2;1 (Golldack et al., 2002; Kader et al., 2006; Horie et al., 2007), and OsHKT2;2 (Kader et al., 2006). All results emphasize that HKT transporter is actively expressed in tissues (e.g., the root epidermis and cortex, xylem and phloem, and vascular bundle region). Expression patterns of subfamily 1 transporters have been consistently found in the vasculature and rarely in other tissues, whereas expression of subfamily 2 transporters has mainly been observed in root periphery cells and often tissues in or near to the plant vasculature (Horie et al., 2007). OsHKT1;1 and OsHKT1;3 are mostly expressed in bulliform cells, enlarged epidermal, cells, and are responsible for both Na^+^ and K^+^ permeability; thus, HKT transporter expression is not restricted to root periphery cells and vascular regions (Jabnoune et al., 2009). Rice has a total of nine HKT transporter genes, which raises interesting questions about the functional diversity within this transporter family (Garciadeblás et al., 2003).

Our result demonstrates differentially induction of *HKT* family genes in the roots and shoots of the four tested rice genotypes, *OsHKT1* was highly expressed in the roots of Cheongcheong and Pokkali and the shoots of Nagdong and Pokkali after 24 and 48 h. Additionally, upregulation *OsHKT3, OsHKT4*, and *OsHKT6*, 7–17-fold increases in expression were found in the roots of Cheongcheong after 24 and 48 h of stress, whereas *OsHKT6, OsHKT7, OsHKT8*, and *OsHKT9* were downregulated in both the roots and shoots of Cheongcheong. In the salt-sensitive cultivar IR28, all *OsHKT* family genes were downregulated in both the roots and shoots (compared with control expression), except for *OsHKT3, OsHKT8*, and *OsHKT9* expression in the roots.

Golldack et al. (2002) previously reported that *OsHKT1* transcription is downregulated in the root tips of Pokkali and IR29 under salt stress. We found a consistent difference in expression of *OsHKT* family genes among the tested rice cultivars; however, high expression of *OsHKT3, OsHKT8*, and *OsHKT9* was observed in the shoots of Pokkali under salt stress while low expression of *OsHKT3, OsHKT7, OsHKT8*, and *OsHKT9* was observed in the roots. The lab cultivar Nagdong showed the highest expression of *OsHKT1, OsHKT2, OsHKT4, OsHKT6*, and *OsHKT8* in the shoot region while expression levels in the roots were lowest (except for expression of *OsHKT7* and *OsHKT8* genes) under salt stress.

In a previous study, OsNHX proteins were placed into two subgroups: *OsNHX1–OsNHX4* and *OsNHX5*. These play important roles in the response to salt stress; for example, *OsNHX1, OsNHX2, OsNHX3*, and *OsNHX5* can suppress Na^+^, Li^+^, and K^+^ that are localized in the tonoplast (Fukuda et al., 2011). Another study reported that *nhx1* mutants exhibit lower K^+^ content in both the shoots and roots compared with that found in the wild type (Pardo et al., 2006). Under stress conditions, transcription levels of *OsNHX1* in the shoots are higher than those in the roots, whereas transcription levels of *OsNHX5* are higher in the roots than those in the shoots (Fukuda et al., 2004, 2011). We also found that expression of *OsNHX* family genes was regulated differentially in the roots and shoots of the four tested rice genotypes. High expression of *OsNHX1* was observed in the roots of IR28 and Pokkali, but expression was higher in the shoots of Pokkali. Similarly, *OsNHX2* was highly expressed in the roots of Nagdong and IR28, whereas expression of this gene in the shoots was found to be higher in IR28 and Pokkali after 8, 24, and 48 h of stress. Zhang et al. (2018) previously reported high expression of *OsNHX3* and *OsNHX4* in the salt-tolerant *JYGY-1* rice variety under salt stress. Our results also indicate that expression of *OsNHX3* and *OsNHX5* was relatively high in roots and shoots after 24 and 48 h of salt stress in the salt-tolerant variety Pokkali. Similarly, in the roots of Nagdong, high expression levels of *OsNHX3* were observed after 24 h of salinity stress, whereas expression of *OsNHX3* and *OsNHX5* was relatively low in the shoots of Cheongcheong after 8 h of salt stress.

The SOS pathway is involved in maintaining the Na^+^/K^+^ ratio in cells; the SOS1 Na^+^/K^+^ antiporter reduces accumulation of Na^+^ and improves salt tolerance in mutant cells (Shi et al., 2002). In *Arabidopsis*, the SOS1 genetic locus confers salt tolerance (Wu et al., 1996). In a previous study, high induction of *OsSOS1* was observed under salt stress in the roots of cultivated and weedy rice (Zhang et al., 2018). In the present study, high expression of *OsSOS1* was observed in the roots and shoots of both Cheongcheong and Nagdong cultivars after 8, 24, and 48 h of salt stress; in contrast, *OsSOS1* was least expression after 24 h in the shoots of salt-tolerant Pokkali. Under salt stress, the high expression of *OsSOS1* in both Cheongcheong and Nagdong might have led to the discharge of toxic apoplastic Na^+^ from inside the cellular environment, which in turn would have led to better salt tolerance management.

### Ion Homeostasis and Combinations Among Rice Genotypes

Various studies have suggested that salt tolerance is ultimately manifested in plants through several physiological processes. During salt stress, high Na^+^ concentrations outside of plant cells create an electrochemical gradient that facilitates the transport of Na^+^ into the cell through K^+^ transporters, resulting in high cytosolic Na^+^ concentrations (Blumwald, 2000). Salt-stressed plants show high Na^+^ contents and this increase in intracellular Na^+^ results in destructive effects caused by competition with K^+^ in enzyme activation and protein biosynthesis (Shabala and Cuin, 2008; Wang et al., 2013). Plants under salt stress show not only increased Na^+^ uptake but also reduced K^+^ uptake (Horie et al., 2001; Zhu, 2003). K^+^ ions are important to plants; by increasing K^+^ content, plants can reduce Na^+^ ions to some extent, which thereby reduces the Na^+^/K^+^ ratio (Zhang et al., 2018). The extent of salt tolerance in plants is known to be correlated with a more efficient system for the selective uptake of K^+^ over Na^+^ (Noble and Rogers, 1992; Ashraf and O'leary, 1996). In the current study, both Na^+^ and K^+^ ions were significantly increased in the roots and shoots of all four rice genotypes when compared with levels in the control. Among treated plant roots, the Na^+^ ratio was higher than that of K^+^ in Cheongcheong and IR28, whereas Nagdong and Pokkali had higher K^+^ than Na^+^ thus it might be possible that cultivars Pokkali and Nagdong had more salt-tolerant than that of Cheongcheong and IR28.

A previous study reported a negative relationship between Mg^+^ and K^+^ ions (Valdez-Aguilar et al., 2009). However, in our study, we measured a decrease in Mg^+^ ions rather than K^+^ ions in the roots and shoots of all four treated rice genotypes. High salt absorption interferes with the absorption of other nutrient ions such as Ca^2+^, K^+^, N, and P, which results in nutritional deficiency and eventually reduced yield and quality (Grattan and Grieve, 1999). We found a significant increase in Ca^2+^ in the roots and shoots of treated Cheongcheong and IR28 cultivars but a decrease in Ca^2+^ in the roots of Nagdong and shoots of Pokkali. A previous study reported that mangrove (*Bruguiera parviflora*) leaves under salt stress cannot alter their endogenous levels of K^+^ and Fe^2+^ (Parida et al., 2004). However, in our study, we found a significant decrease in Fe^2+^ in the roots of Cheongcheong and Pokkali and in the shoots of IR28, in contrast to an increase in Fe^2+^ in the roots and shoots of Nagdong. Similarly, Fe^2+^ was increased in the roots of IR28 and shoots of Cheongcheong and Pokkali. Achakzai et al. (2010) reported that sunflowers subjected to high doses of salinity increase their uptake of micronutrients including Cu^2+^, Mn^2+^, and Fe^2+^ in the roots, and Cu^2+^, Fe^2+^, and Zn^2+^ in the shoots. Salt stress has also been shown to increase the concentration of Na^+^, Ca^2+^, Mg^2+^, and Cu^2+^ while decreasing the concentration of P and K^+^ in the shoots and straw of IR28 and IR101998-66-2 (Verma and Neue, 1984). In our study, Zn^2+^ uptake decreased in the roots of Cheongcheong and the roots and shoots of Pokkali under salt stress. On the other hand, Zn^2+^ uptake increased in the roots and shoots of Nagdong and IR28 and the shoots of Cheongcheong. Salinity stress, also increased Cu^2+^ uptake in all four genotypes (except in the shoots of IR28, in which a slight reduction was observed). Mg^2+^ uptake increased in the roots and shoots of all salt-stressed rice genotypes, except in the roots of Cheongcheong. Finally, Mn^2+^ uptake increased in Cheongcheong, Nagdong, and IR28 roots and shoots but decreased in the roots of Nagdong and roots and shoots of Pokkali.

Given that rice is a staple food facing yield reduction issues caused by environmental stress, climate change, and geographic problems, there is a need to understand how rice cultivars respond to salinity stress. In the present study, Pokkali and Nagdong cultivars were found to have strong salt tolerance during both seed germination and seedling growth. Under 150-mM NaCl stress, these cultivars showed slight reductions in plant growth, but the plants remained vigorous in comparison to Cheongcheong and the salt-sensitive cultivar IR28. The salt-tolerant varieties Pokkali and Nagdong enhance their salt tolerance by regulating the Na^+^/K^+^ ratio in the roots and shoots regions. Under salt stress, ion homeostasis is considered a complex network system that regulates other micronutrients together with *OsHKT, OsNHX* family, and *OsSOS1* genes. Our results indicate that ion transport-related genes and other micronutrients are differentially regulated among rice cultivars under salt stress. In conclusion, growth of the plant molecular breeding lab cultivar Nagdong is superior to that of Cheongcheong under salt stress. The findings of the present study could contribute to the development of high-yielding and salt-tolerant varieties of rice for plant molecular breeding via the CRISPR/Cas9 system.

## Data Availability Statement

The raw data supporting the conclusions of this article will be made available by the authors, without undue reservation.

## Author Contributions

MF planned, designed and performed the research, and analyzed the data and wrote the findings. J-RP contributed to experimental resources. Y-HJ and E-GK contributed to the ICP and statistical analysis. K-MK edited the manuscript. All authors read and approved the final manuscript.

## Conflict of Interest

The authors declare that the research was conducted in the absence of any commercial or financial relationships that could be construed as a potential conflict of interest.

## Publisher's Note

All claims expressed in this article are solely those of the authors and do not necessarily represent those of their affiliated organizations, or those of the publisher, the editors and the reviewers. Any product that may be evaluated in this article, or claim that may be made by its manufacturer, is not guaranteed or endorsed by the publisher.
